# Survey of feature selection and extraction techniques for stock market prediction

**DOI:** 10.1186/s40854-022-00441-7

**Published:** 2023-01-12

**Authors:** Htet Htet Htun, Michael Biehl, Nicolai Petkov

**Affiliations:** Bernoulli Institute for Mathematics, Computer Science, Artificial Intelligence, University of Groningen, Groningen, The Netherlands

**Keywords:** Feature selection, Feature extraction, Dimensionality reduction, Stock market forecasting, Machine learning

## Abstract

In stock market forecasting, the identification of critical features that affect the performance of machine learning (ML) models is crucial to achieve accurate stock price predictions. Several review papers in the literature have focused on various ML, statistical, and deep learning-based methods used in stock market forecasting. However, no survey study has explored feature selection and extraction techniques for stock market forecasting. This survey presents a detailed analysis of 32 research works that use a combination of feature study and ML approaches in various stock market applications. We conduct a systematic search for articles in the Scopus and Web of Science databases for the years 2011–2022. We review a variety of feature selection and feature extraction approaches that have been successfully applied in the stock market analyses presented in the articles. We also describe the combination of feature analysis techniques and ML methods and evaluate their performance. Moreover, we present other survey articles, stock market input and output data, and analyses based on various factors. We find that correlation criteria, random forest, principal component analysis, and autoencoder are the most widely used feature selection and extraction techniques with the best prediction accuracy for various stock market applications.

## Introduction

Financial time-series prediction is an attractive research area for investors, market analysts, and the general public because it offers opportunities to increase wealth. In financial markets, various assets such as stocks, bonds, currencies, and commodities are traded at prices determined by market forces. Among the different assets, equities are the most interesting with respect to the prediction of short- or long-term market prices, returns, and portfolio management. Stock market analysis includes two major schools of thought: technical and fundamental analysis. Technical analysis forecasts the development of stock prices through an analysis of historical market data, such as price and volume. A large part of the literature (Nazario et al. 2017; AI-Shamery and AI-Shamery 2018; Lahmiri 2018; Lin et al. 2021; Lin 2018; Sugumar 2014; Picasso et al. 2019) is focused on technical analysis based on technical indicators to identify the movement direction of stock prices and turning points in the time series. Different types of technical in- dicators, such as stochastic oscillator, moving averages, and relative strength index (RSI), are used in prediction models, and the effectiveness of these input features for future stock market forecasting is studied.

Fundamental analysis uses economic indicators related to firm performance and the state of the economy. In Kohli et al. (2019), for example, macroeconomic factors, such as com- modity prices, market history, and foreign exchange rates, were used to forecast the direction of the Bombay Stock Exchange. Chen et al. (2017) applied financial indicators to select the optimal stocks from the Taiwan stock market. A combined analysis of technical and fundamental indicators was conducted in Nti et al. (2020a), Thakkar and Chaudhari (2021) by using various artificial intelligence algorithms. These theories are challenged by the widely accepted random walk hypothesis (Fama 1995) and efficient market hypothesis (Malkiel 2003), which suggest that future changes in stock prices cannot be predicted from the historical data as fluctuations are independent and random. Therefore, future stock price changes are widely known to be unpre-dictable. However, many financial economists, researchers, and traders believe that stock prices are at least partially predictable because price changes tend to repeat themselves owing to the collective and patterned behavior of investors (Zhang et al. 2018).

As machine learning (ML) techniques and computer resources have become more widely available, numerous statistical, ML, and deep learning (DL) methods have been deployed in stock market forecasting (Gandhmal and Kumar 2019; Shah et al. 2019). Some of these methods are described below.

### Statistical methods

The autoregressive integrated moving average (ARIMA), one of the most effi- cient and robust statistical models, was applied to predict daily stock returns and prices in Jarrett and Schilling (2008); Khan and Alghulaiakh 2020). An ARIMA model has also been combined with other methods, such as XGBoost, wavelet transform, and neural network models (Wang and Guo March 2020; Shan et al. 2015), to predict the one-day-ahead open prices of different stocks. The authors demonstrated that hybrid models achieve better performance than a single model for stock market predictions. In Ho et al. (1988), a hybrid method of ARIMA with a neural network and long short-term memory (LSTM) network was applied to predict the Bursa Malaysia stock exchange during the COVID-19 pandemic period.

### ML methods

In Lahmiri (2014), Hu et al. (2013), Nti et al. (2020b), Yu and Liu (2012), support vector machine (SVM), a popular ML method, was suc- cessfully deployed for regression and classification tasks using technical indicators and macroeconomic factors. The SVM method also provided good prediction performance for high-frequency data in Henrique et al. (2018). Tree-based ensemble methods (Basak et al. 2019; Weng et al. 2018) are also popular for stock price prediction owing to their low variance. Random forest (RF) is an ensemble method that provides satisfactory prediction results for stock direction (Sadorsky 2021) and stock selection (Tan et al. 2019) using common technical indicators.

### DL methods

Several recent studies have addressed stock market trend forecasting using DL neural networks to extract the essential characteristics of highly complex stock market data. In Guresen et al. (2011), Ruxanda and Badea (2014), Selvamuthu et al. (2019), the authors applied an artificial neural net- work (ANN) to predict the stock market index, stock price direction, and tick-by-tick data. A study (Selvin et al. 2017) applied three DL models to predict the prices of National Stock Exchange (NSE)-listed companies in India and used a slid- ing window approach for short-term predictions. In Xu et al. (2018), a recurrent neural network (RNN) model was applied to predict the up or down direction of stocks on the basis of financial news and historical stock prices. Kumar et al. (2021a) and (2021b) deployed an RNN classifier for intraday stock market prediction, analyzed relevant technical indicators and identified a hidden pattern of stock trends by using a recursive feature elimination (RFE) method.

With the increase in the number of different types of features in the stock market, feature selection techniques have been widely used in conjunction with predictive models in a variety of stock market applications. These features include daily stock information (open, high, low, close, volume (OHLCV) data), technical and economic indicators, and financial news. In Botunac et al. (2020), Tsai and Hsiao (2010), Ni et al. (2011), the application of a feature selection method was found to produce more effective predictions than the use of prediction models alone. Therefore, various feature selection techniques that are applied in the stock market and their specific performance must be reviewed to further improve predictions.

### Importance of feature selection process

**Fig. 1:**
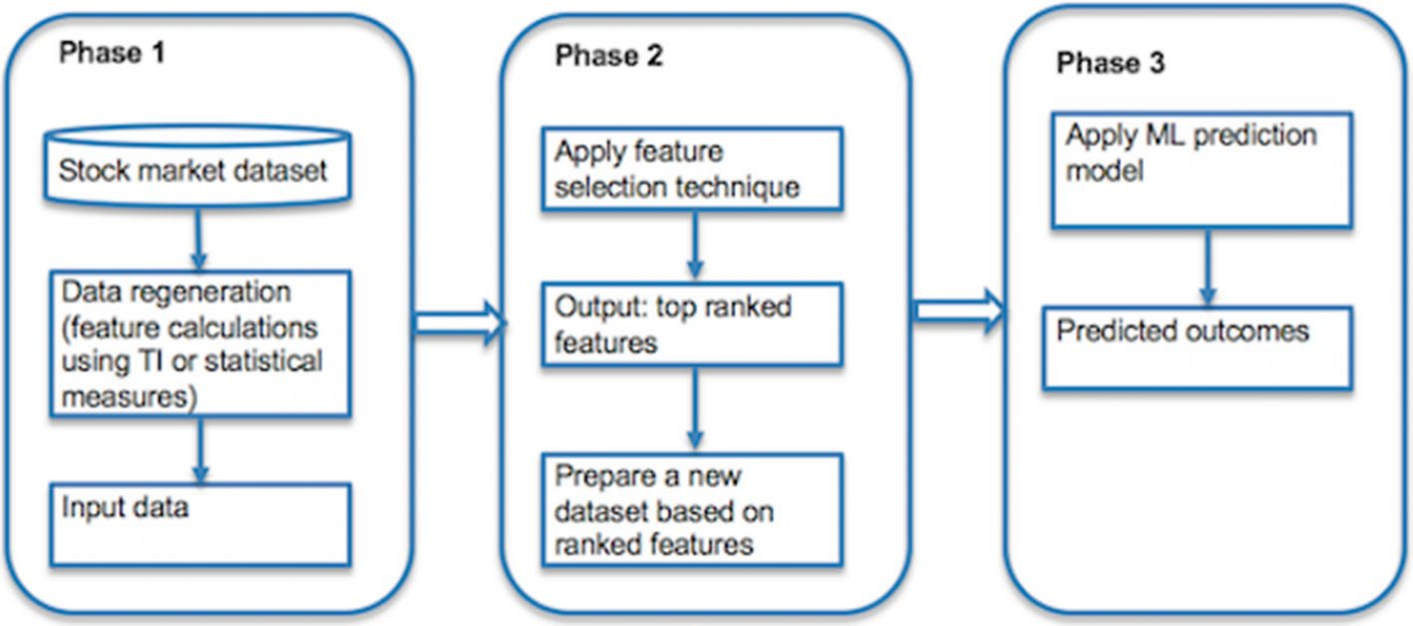
Phases of the stock market prediction with feature selection and ML method combination

In stock market analysis, price changes are influenced by many factors, such as historical stock market data, fundamental factors, and investors’ psychological be- haviors. The diversity of features presents a challenge in achieving higher prediction accuracy. Thus, a feature selection process should be performed to select key fea- tures from the original feature set before applying an ML model to predict outcomes. The feature selection process also helps to reduce irrelevant variables, computational cost, and the overfitting problem and improves the performance of ML models (Cai et al. 2012). If we select only a small number of features as input for an ML model, the in- formation may not be enough to make predictions. A large number of features also increase the running time and causes the generalization performance to deterio- rate owing to the curse of dimensionality (Kim 2006). Therefore, only the most significant features that affect the results should be selected to achieve successful predictions. The current survey article presents various types of feature selection techniques and their different criteria for the selection of the relevant features of stock data. Figure 1 illustrates the flow diagram of the feature selection process combined with ML methods for the prediction of stock market data.

### Survey method

We collected research articles published in the last 12 years (2011–2022) through a keyword search performed on July 8, 2022. The following terms were used to search article titles, abstracts, and keywords from two scientific databases, namely, Scopus and Web of Science:

((“stock market”) AND (“prediction” OR “forecasting”) AND (“feature selec- tion” OR “feature study” OR “feature extraction” OR “feature learning” OR “fea- ture generation” OR “feature engineering” OR “feature representation” OR “fea- ture fusion” OR “feature reduction” OR “feature weighted” OR “feature analysis”)).

**Fig. 2:**
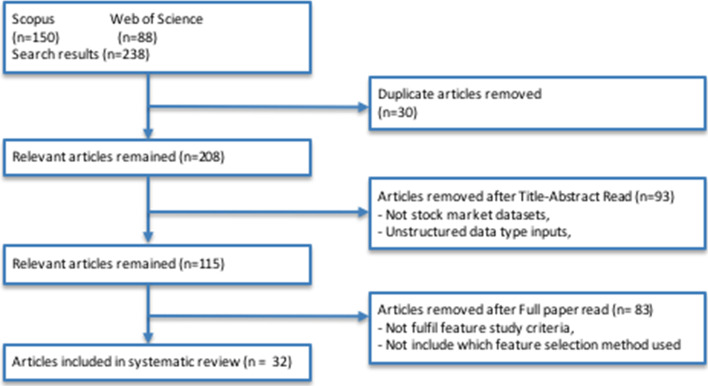
Flow diagram of the selected articles

The results were restricted to the following research areas: computer science, information systems; computer science, theory and methods; economics; business economics; business management and accounting; mathematics; computer science; engineering; engineering, electrical and electronic; computer science, interdisciplinary applications; computer science, artificial intelligence; decision sciences; and social sciences. Moreover, this survey focused on studies that used structured-type inputs: OHLCV data, technical indicators, and fundamental indicators in the stock market. Thus, articles that applied unstructured inputs, such as text from news, social net- works, and blogs, were not included. A total of 238 articles were selected from both databases, and 30 articles were found to be duplicates. After reading the titles and abstracts of the remaining 208 articles, we removed 93 articles that used unstruc- tured inputs, leaving 115 articles. Subsequently, we excluded 83 articles that did not mention the feature selection methods applied. Therefore, we obtained 32 relevant papers (27 in journals (Alsubaie et al. 2019; Aloraini 2015; Li et al. 2022; Kumar et al. 2016, 2021b; Nabi et al. 2019; Yuan et al. 2020; Shen and Shafiq 2020; Haq et al. 2021; Sun et al. 2019; Chen and Hao 2017, 2020; Gunduz et al. 2017; Siddique and Panda 2019; Singh and Khushi 2021; Ampomah et al. 2020, 2021; Qolipour et al. 2021; Das et al. 2019; Tang et al. 2018; Chong et al. 2017; Bhanja and Das 2022; Xie and Yu 2021; Dami and Esterabi 2021; Gunduz 2021; Barak et al. 2017; Farahani and Hajiagha 2021), and 5 in conference proceedings (Botunac et al. 2020; Cai et al. 2012; Labiad et al. 2016; Rana et al. 2019; Iacomin 2015). Figure 2 illustrates the article selection method.

This survey aimed to answer the following research questions:

1. Which types of feature selection and extraction techniques are applied in stock market prediction?
2. Which structured inputs are widely used in prediction models?
3. How can a feature learning process improve prediction accuracy?

### Related work

This section describes existing survey articles related to stock market prediction. Most review papers discuss the applicability of various ML, ensemble learning, and DL methods.

Different types of prediction models (support vector regression (SVR), neural network-based models) and clustering techniques (k-means, fuzzy, optimization) were analyzed in Gandhmal and Kumar (2019) on the basis of the types of methods, datasets, performance measures, and software tools. In Henrique et al. (2019), a bibliometric analysis was performed to re- view common ML techniques applied in financial markets from 1991 to 2017. Fore- casting methods such as ARIMA, SVM, decision trees, and neural networks were applied in Henrique et al. (2019) to predict the prices, directions, returns, and volatility of different stock markets. A recent survey (Bustos and Pomares-Quimbaya 2020) covering 2014–2018 classified articles according to the type of input variables. Another extensive and comparative analysis of en- semble techniques was conducted in Nti et al. (2020c) to predict the 30-day-ahead closing prices of four market indices.

In Ican and Celik (2017), ANN models were reviewed for the directional predictions of the stock market, and different studies were compared in terms of the input features, time span of prediction, and forecasting performance. Kumar et al. (2021a) reviewed 30 research papers and concluded that ANN models are the most widely used method in various stock market applications. In addition, they concluded that some hybrid models achieve better accuracy for financial time-series predictions.

In Sezer et al. (2020), the authors studied DL models, convolutional neural networks (CNNs), deep belief networks, RNNs, LSTM, and deep reinforcement learning and concluded that LSTM is the most frequently used model in stock market prediction because of its clear model creation and higher performance for time series data. Nine deep neural networks (DNNs) were presented in a survey of DL methods for stock price and trend prediction (Thakkar and Chaudhari 2021). The authors also provided comparative experiments of various DNN models based on a number of different features for five-day-ahead trend predictions; a deep Q-network model obtained the highest average directional accuracy regardless of the number of features. In Kou et al. (2021), the authors applied four feature selection methods to identify the optimal subset of features to be used in bankruptcy predictions for small and medium-sized enterprises. They discussed the significance of the feature selection process for improving the performance of prediction models. A review study (Kou et al. 2020) evaluated several filter feature selection methods for the binary and multiclass classification of text datasets. On the basis of several evaluation criteria, including classification performance, stability, and efficiency, the authors presented the document frequency feature selection method as the most recommended approach. We observed that a limited number of feature selection methods are provided in existing empirical and survey papers and that not all types of feature selection and extraction techniques are addressed.

### Data inputs and prediction outputs

We focused on structured-type inputs, which are mainly used as features in various stock market applications, because their information is systematic and the processing techniques are well-defined. Three main types of structured inputs are used in stock market prediction: basic features, technical indicators, and fundamental indicators.

(i) *Basic features* are stock values such as OHLCV data; closing prices are the most commonly used information to predict the prices of the next trading day.
(ii) *Technical indicators* are extracted from historical price series using mathe- matical formulae and are used to analyze the particular patterns of past prices and predict future movements. The most common technical indicators (Alsubaie et al. 2019) are the RSI, stochastic oscillator, and moving average convergence-divergence. Some studies such as Botunac et al. (2020) and Qolipour et al. (2021), used a combination of basic features and technical indicators to forecast stock market direction.
(iii) *Fundamental indicators* are economic indicators (Bustos and Pomares-Quimbaya 2020) ranging from macroe- conomic factors, such as a country’s or government’s overall economic status, to microeconomic factors, such as the information of an individual company. Macroe- conomic factors, such as interest rates, consumer price index, and the overall state of the economy, are the most commonly used fundamental indicators. Forecasting based on fundamental indicators is less common in the literature because of the difficulty in building models that explain why a stock’s price fluctuates.

In terms of the outputs from learning models, the two target predictions are value/return and the direction of the stock. Predicting value/return is a regression task while predicting direction (up or down) is a classification task.

The remainder of this paper is organized as follows. Section 2 describes the differ- ent feature selection methods, and Section 3 reviews the feature extraction methods combined with various ML models for different target variables. Section 4 discusses the analyses based on different factors, and Section 5 provides the limitations and future directions. Finally, Section 6 presents the conclusions of the study.

## Feature selection methods

Under dimensionality reduction, two approaches can be used: feature selection and feature extraction. They are basically the same approach, but they differ in their approaches to selecting useful and reducing irrelevant features. Feature selection maintains a subset of the original features, whereas feature extraction creates new features from the original dataset.

The feature selection process delivers only unique features that contribute the most to the prediction outcomes by removing noise and irrelevant features. This section presents a review of different feature selection methods applied to stock market predictions. These methods are categorized into four types: filter, wrapper, embedded, and information theory-based methods.

### Filter methods

Filter methods rank variables according to their relevance to the underlying ML algorithms. They act as a preprocessing step by selecting highly ranked features and applying them to ML methods (Urbanowicz et al. 2018). Therefore, they are computationally fast and robust to overfitting but ignore the dependency between features. Filter methods use statistical performance measures such as the correlation/distance between features and output variables.

#### Correlation and distance criteria

The correlation coefficient, such as the Pearson correlation coefficient (PCC) and Spearman rank correlation, is the simplest way to calculate the relevance score be- tween a feature and a target variable (*f, t*). Aloraini (2015) applied the Pearson and Spearman correlations as part of the ensemble feature selection process to rank 11 features, which are the daily open prices of 11 stocks. They combined univariate methods with other feature selection methods to identify hidden relationships be- tween predictors. Their empirical experiments revealed that the proposed ensemble feature selection method achieved better predictive results than single feature se- lection methods. In another study, Li et al. (2022) applied PCC to select features with a PCC value of 0.5 as input data to a broad learning system (BLS) model for one- day-ahead closing price prediction. On the basis of 11 years of experimental data for stocks from the Shanghai Stock Exchange, they stated that the proposed method, which combines PCC and BLS, outperformed 10 previous single ML methods.

In Kumar et al. (2016), linear correlation (LC) and rank correlation (RC) methods were deployed together with a proximal support vector machine (PSVM) model as the LC-PSVM and RC-PSVM to obtain the optimal feature subset from an original set of 55 tech- nical indicators for 12 different stock indices. Two studies, (Alsubaie et al. 2019) and (Nabi et al. 2019), also used an LC method with different classifiers to predict the direction of stock markets.

The Euclidean and Manhattan methods calculate the distance between any two data points (*f, t*), where f is the examined feature and t is a target variable in the feature space. In (Aloraini 2015), distance-based measures were applied to solve the feature selection process and combined with an ML method for daily open price predictions.

#### Relief algorithm

The relief algorithm (Kira and Rendell 1992) is used for feature selection in regression and classification problems. This algorithm calculates the importance score for each feature on the basis of how well the feature can distinguish between nearest-neighbor instances. It returns a ranked list of features or top-scoring features based on a given threshold. Kumar et al. (2016) proposed hybrid prediction models that combine feature se- lection techniques and an ML model (PSVM). They applied the regression relief (RR) algorithm as a feature selection method and compared it with other feature selection methods, including LC, and RC methods. The results of their study of the one-day-ahead direction of 12 stock indices revealed a negligible difference between the performance of the RR and correlation-based feature selection methods. Another study (Alsubaie et al. 2019) applied a relief algorithm to select highly ranked features from 50 common technical indicators for large datasets, which included 99 stocks and 1 market index. They tested the performance of feature selection methods on the basis of two categories: accuracy- and cost-based criteria. The relief algorithm was the best-performing filter in the accuracy and cost-based evaluations. They concluded that selecting more than 30 technical indicators is likely to reduce the classification performance for their datasets.

The relief method was also used in a study (Gunduz et al. 2017) that selected 25 indicators of daily stock prices for the three most traded stocks in the Borsa Istanbul (BIST) stock market with the gradient boosting machine (GBM) classifier. The authors then evaluated the performance of the relief algorithm with a different gain ratio approach and concluded that the accuracy values for the applied stocks were similar for both feature selection techniques.

### Wrapper methods

In wrapper methods, feature selection is wrapped within the learning process of an ML algorithm. Hence, these methods look for a subset of features that provide the highest prediction performance. They also rely on the performance of the predictor to obtain an optimal feature subset and use the accuracy of this predictor as the ob-jective function. Wrapper methods are known for being computationally expensive because of the large number of computations (multiple rounds of training) required to obtain the critical feature subset and address the overfitting problem.

#### Recursive feature elimination (RFE)

RFE (Guyon et al. 2002) is a well-known wrapper-type feature selection technique that involves an iterative procedure to train an ML model. RFE computes the ranking criterion for all features in each training and removes the features with the lowest importance score; then, it trains the model again on the basis of the new feature set.

The RFE technique has been used in several studies for various stock market applications. Yuan et al. (2020) applied an RFE algorithm based on an SVM model to achieve a proper feature subset from 60 features of 10 different categories for predicting all stocks in the Chinese A-share stock market. The authors used the SVM-RFE method to retrieve the importance scores of all 60 features and then chose the top 80% of the features (i.e., 48 features) as input features for the SVM, RF, and ANN models to predict the direction of monthly stock returns. In Botunac et al. (2020), RFE was proposed as a feature selection method to find the effective features from five basic features and nine technical indicators of various stocks for the LSTM fore- casting model. As RFE generated unclear scores for all features in the preliminary experiments, the authors also applied other feature importance methods, such as linear regression, decision tree, and RF regression. Another study (Shen and Shafiq 2020) applied RFE to explore the most effective features in the feature space. The authors designed an RFE algorithm to remove one feature at each step and selected all relevant and effective features to build a good predictive model with an LSTM network.

### Embedded methods

Embedded methods combine the qualities of filter and wrapper methods and form feature selection as part of the training process by simultaneously integrating al- gorithm modeling and feature selection (Urbanowicz et al. 2018). Therefore, they are more computa- tionally efficient and suffer less from overfitting than wrapper methods. Embedded and wrapper methods are considered as subset evaluation techniques that can capture dependencies and interactions between features. This capability makes these methods superior to filter methods.

#### Random forest (RF)

RF (Breiman 2001) is an ensemble learning method used for both classification and regression problems. It uses a bootstrapped aggregation technique and a random selection of features to construct each decision tree in a forest. It combines the simplicity of individual decision trees and outputs the mode of the classes for classification and the mean prediction for regression based on multiple decision trees. It is widely ap- plied owing to its favourable characteristics, such as good generalization, simplicity, robustness, and low variance.

Recently, RF has been increasingly exploited as a feature selection method because it has many advantageous qualities, such as internal estimates of error, correlations, and feature importance scores. RF provides two methods for calculating feature importance scores: mean decrease accuracy (MDA) and mean decrease impurity (MDI) (Labiad et al. 2016). MDA describes how much prediction accuracy the model loses after removing each feature, and MDI is a measure of how each feature contributes to the homogeneity of the nodes and leaves for each decision-tree model. Therefore, the larger the value, the higher the importance of the feature for the MDA and MDI methods.

RF is a feature selection method that has been applied in various stock market prediction studies. Haq et al. (2021) deployed the MDA method to generate optimal feature subsets from a large set of 44 technical indicators. The authors also used two other feature selection methods, namely, logistic regression (LR) and SVM, and selected 20 identical features by using the three feature selection techniques. Accord- ing to their evaluation measures, classification accuracy, and Matthews correlation coefficient, they indicated that combined features selected by multiple disjoint tech- niques provided higher accuracy for the prediction model than the features selected by a single feature selection technique.

The authors of Kumar et al. (2016) applied RF to remove redundant and highly correlated vari- ables from 55 technical indicators and used the PSVM model to predict the one- day-ahead direction of 12 different indices from international markets. To evaluate the performance of the RF feature selection technique, they applied three other fea- ture selection methods and observed that RF-PSVM is the only hybrid model that achieves higher accuracy than the individual PSVM for all datasets. Furthermore, the results showed that the RF method can suggest a certain number of indicators that provide better prediction results than other feature selection methods. In Botunac et al. (2020), RF was also utilized to determine the importance scores of 14 features to predict the closing prices of Apple, Microsoft, and Facebook. Another research (Yuan et al. 2020) proposed RF as a feature selection method and a prediction model (RF-RF) to perform stock price trend prediction; the proposed approach achieved the best performance among all the integrated models in the study. In Labiad et al. (2016), RF was applied to assess the impor- tance of each input variable using MDI and MDA for feature selection to classify the direction of 10-min-ahead prediction. Therefore, existing research papers in- dicate that RF achieves satisfactory predictions as a feature selection technique and as a prediction model and delivers superior performance over other types of feature selection methods.

In Rana et al. (2019), ensemble learning approaches such as the decision-Tree classifier and extra- trees classifier were deployed to select important predictors from basic features (OHLCV data); the experiment results revealed that the closing price is the most significant feature.

#### Other embedded methods

In some studies, other embedded methods, such as SVM and LR models, have been applied as feature selection techniques to identify proper feature subsets as inputs to deep generative models (Haq et al. 2021). Another study (Aloraini 2015) used the lasso estimation for feature selection and regularization processes to select the best subset of predictors for each bank in the Saudi stock market. In Cai et al. (2012), a restricted Boltzmann machine (RBM) was applied as a feature extractor. The RBM (Smolensky 1987) is a type of energy-based model and a special case of general Boltzmann machines based on hidden units in the machine; the extracted features are determined by the expected value of the hidden units of a learned RBM.

### Information theory-based methods

Information theory-based methods utilize mutual information (MI) to obtain the importance score of each feature; examples of these methods include the forward selection minimal-redundancy-maximal-relevance (FSMRMR) (Peng et al. 2005) and conditional mutual information maximization (CMIM) (Nguyen et al. 2014) methods. In Sun et al. (2019), the authors applied the FSMRMR method, which considered the combination of two measures (relevance and redundancy of the features) using average bivariate MI, and the CMIM method, which considered the redundancy and interaction of the features as a higher priority. The FSMRMR and CMIM methods were combined with the learning model ARMA-GARCH to prognosticate intraday patterns for market shock direction. The authors indicated that the FSMRMR method can lead to a consid- erably higher performance in terms of accuracy rate and root mean squared error than the CMIM method.

Chen and Hao (2017) used the information gain method, which is an attribute se- lection approach based on the number and size of branches in a decision learning system, to estimate the relative importance of each attribute. Using the information gain method, the authors constructed a feature weighted matrix of nine technical in- dicators, which were inputs in the SVM and k-nearest neighbor (KNN) algorithms.

The performance of these models was evaluated for two Chinese stock market in- dices to predict 1-, 5-, 10-, 15-, 20-, and 30-day-ahead prices. The article cited in Chen and Hao (2020) also applied the information gain method to measure the importance of technical indicators used to predict buy and sell signals for 30 Chinese stocks. The authors reported that a prediction model using a feature weighted SVM and an informa- tion gain approach achieves higher accuracy than a prediction model without any feature selection.

A modification of the information gain method, the gain ratio approach, was applied in Alsubaie et al. (2019) to rank 50 technical indicators for the application of investment return prediction and a trading strategy using nine different classifiers. The results showed that the best Sharpe ratios, which determine the balance between investment re- turn and risk, were achieved on the basis of only the top 5 or 10 technical indicators for most classifiers. Another study (Gunduz et al. 2017) used the gain ratio method to select tech- nical indicators for the GBM prediction model. On the basis of the results, the authors demonstrated how feature selection improved the daily return predictions for applied stocks from the BIST stock market.

## Feature extraction techniques

Feature extraction methods reduce the number of features in a dataset by creating new features that summarize most of the information contained in the original set of features. Two types of feature extraction techniques were identified in the reviewed studies: statistical and optimization-based techniques.

### Principal component analysis

Principal component analysis (PCA) (Jolliffe 2002), which is a statistical-based feature ex- traction method, is the most popular technique for dimensionality reduction. It transforms a high-dimensional feature vector into a low-dimensional feature vec- tor with uncorrelated components by calculating the eigenvectors of the covariance matrix of the original features. Therefore, PCA is simple to implement and versatile. Among the 32 reviewed papers, 11 studies used PCA to identify the most relevant features for the learning models. The authors in Siddique and Panda (2019) applied a hybrid forecasting model, SVR-particle swarm optimization (PSO) combined with PCA, to remove the least influential features from the original 35 ones to predict the next-day closing prices of the Tata Motors stock index. Empirical experiments with and without PCA clearly showed that the PCA-SVR-PSO model with the 11 features extracted by PCA gives lower error values than the SVR-PSO model in all evaluation criteria: mean absolute error (MAE), root mean square error (RMSE), and mean absolute percentage error. Singh and Khushi (2021) also applied the PCA method to identify a smaller set of features that were the top contributors in the model from the original 28 features. They demonstrated that a reduced subset of six features produced accuracies similar to those of the original 28 features.

Some studies (Ampomah et al. 2020) and (Qolipour et al. 2021) used PCA to reduce the set of basic features and technical indicators and combined PCA with tree-based ML classifiers to predict the direction of stock returns and price movements. On the basis of confusion matrix evaluation criteria, the authors concluded that ensemble learning models with feature extraction perform better than single learning models. Iacomin (2015) applied the PCA method in combination with the SVM prediction model to forecast the prices of 16 stocks from Bloomberg using 10 common technical indicators. The au- thors demonstrated that the PCA-SVM model outperformed the SVM model for the datasets used.

In (Shen and Shafiq 2020), Shen and Shafiq proposed a complete feature engineering procedure by combining max–min scaling, polarizing for feature extension, RFE for feature selection, and PCA for dimensionality reduction; they tested their approach on 3,558 stocks from the Chinese stock market for short-term prediction. The results revealed that the proposed solution achieved an overall accuracy score of 0.93 and precision and recall scores equal to 0.96 owing to the utilization of different feature engineering approaches combined with the LSTM model. The study in Ampomah et al. (2021) also applied PCA together with feature scaling techniques, namely, standardization and min–max scaling, to find the optimal feature set from 40 technical indicators to predict the direction of seven stocks from the NYSE, NASDAQ, and NSE markets. Another study (Nabi et al. 2019) applied nine different feature selection algorithms combined with 15 different classifiers to predict the monthly direction of 10 companies from NAS- DAQ. As a simple and efficient algorithm, PCA was found to be the best feature extraction algorithm, providing the highest accuracy for all combinations with ML models and different stocks according to the experiments.

Different feature extraction methods were used in Das et al. (2019). The PCA method was com- bined with three neural network-based models: extreme learning machine (ELM), online sequential extreme learning machine (OSELM), and recurrent back propa- gation neural network (RBPNN). They reduced the input of 16 technical indicators and predicted the 1-, 3-, 5-, 7-, 15-, and 30-day-ahead prices for four stock market indices. The empirical results indicated that PCA-ELM and PCA-RBPNN provide better performance in 1-day-ahead prediction than in other days-ahead prediction for all datasets. With respect to the BSE index, the PCA-ELM and PCA-OSELM models are better than the PCA-RBPNN model. PCA was used in the work cited in Kumar et al. (2021b) to extract the features of the ANN prediction model. According to the ex- perimental findings, PCA reduced the complexity and computational cost of the prediction model from the original 20 feature sets to 9 features to predict the clos- ing prices of the Nifty 50, Sensex, and S&P 500 stock indices. The study in Tang et al. (2018) applied PCA for dimensionality reduction to provide information-rich features for a KNN model to forecast the relative returns of 10 indices from the Chinese CSI 300 market. For the Telecom Svc index, the method achieved the highest hit rate of 79.60%.

### Autoencoder

A neural network-based unsupervised learning model called the autoencoder (AE) (Kramer 1991) reconstructs inputs to the neural network in the output layer. The encoder and decoder are its two components. The encoder reduces the input to a codeword-sized dimension, and the decoder uses that codeword to reassemble the original input data.

The study in Chong et al. (2017) applied an AE method to transform raw returns before using them as input in a DNN method to predict the future returns of 38 stocks from the Korean stock market. They created a two-class classification problem based on the upward and downward movements of future returns. According to four evaluation measures, namely, normalized mean squared error (NMSE), RMSE, MAE, and MI, the DNN model with AE outperformed the linear autoregressive model, AR(10), in the test set for 14 stocks with NMSE values smaller than 1. Another study (Bhanja and Das 2022) deployed a CNN-based AE with a series of one-dimensional convolutional and deconvolutional layers for the encoder and decoder. The authors demonstrated that the ML classifiers with the CNN-based AE approach achieved over 80% accuracy for the single-step and multi-step ahead predictions of the S&P BSE SENSEX and Nifty 50 stock market index datasets. Xie and Yu (2021) applied the convolution- based autoencoder (CAE) method to select distinct financial and economic features for the daily direction (up and down) prediction of different stock market indices. They concluded that the average accuracy of the CAE method was approximately 3% higher than that of other methods (i.e., DNN, LSTM, SVM, and PCA) for selected stock indices.

On the basic of the basic (OHLCV) features from the last 10 days, Dami et al. (2021) used an AE with an LSTM model to predict the stock returns of 10 companies from the Tehran Stock Exchange. They showed that in most cases, the performance of the LSTM model with the AE was better than that of the model without the AE. The authors in Gunduz (2021) applied variational autoencoders (VAEs), which are generative AE models, and used a different loss function with AE in network training to choose technical indicators. They used the VAE to forecast the hourly direction of eight banks listed in the BIST 30 index. The authors concluded that models trained with VAE-reduced features had similar accuracy rates to those trained without dimensionality reduction for the selected stocks based on accuracy and F-measures.

Other feature extraction methods.

Linear discriminant analysis (LDA) (Mclachlan 2004) is another feature extraction technique that maximizes the significance of the distance between data points of different categories. The data points of the same class are more compact, and the groups are the most separated from each other. In (Ampomah et al. 2021), the LDA approach was combined with the predictive Gaussian naive Bayes (GNB) model to select the best features from the original set of 40 technical indicators. The authors demonstrated that the predictive model based on the integration of GNB and LDA outperformed other models in their study in terms of accuracy, F1 score, and area under the curve evaluation measures.

The authors in Das et al. (2019) and Ampomah et al. (2021) used factor analysis, another statistical-based feature extraction approach, to achieve significant features for their predictive models. In Das et al. (2019), they used three other optimization-based feature extraction methods: genetic algorithm (GA), firefly optimization (FO), and a combination of FO and GA. They concluded that all the studied feature extraction methods reduced the number of features to obtain better results; the integrated FO and GA method, in particular, displayed outstanding performance with the OSELM prediction model relative to the other feature reduction and prediction methods. Another study (Barak et al. 2017) implemented a prediction model, ANN combined with GA, to extract the best indicators of five stock indices: DAX, S&P 500, FTSE100, DJI, and NDAQ. On the basis of the MAE criterion, the authors compared the performance of the hybrid GA-ANN model with the ARIMA time series model. The study in Farahani and Hajiagha (2021) also developed a GA to select representative features for three classifiers to forecast the returns of 400 companies listed on the Tehran Stock Exchange. An overall accuracy of over 80% was achieved using the selected 15 features from the original 45 features defined by the GA, demonstrating the importance of the feature selection process in predicting stock returns.

## Analysis and discussion

The reviewed articles studied diverse prediction models, feature selection tech- niques, types of features, target predictions, datasets, and evaluation criteria. Table 1 presents a summary of the reviewed papers, and Table 2 compares how well the reviewed studies were performed based on the target predictions and specified evaluation measures. Moreover, our review revealed that feature selection and ex- traction techniques helped obtain better predictions over periods of 10 min up to 1 month ahead in terms of absolute price or direction. Therefore, ignoring fea- ture selection in stock market analysis can have negative effects, such as overfitting, which is likely to damage the overall prediction results of a given learning model.

**Table 1:** Analysis based on types of features, feature selection/extraction techniques, predictive models, and datasets

Study	Types of features	Feature selection/extraction techniques	Prediction methods	Datasets
1. Haq et al. (2021)	Basic features, Technical indicators	LR, SVM, RF	Deep generative model	88 stocks from NASDAQ
2. Labiad et al. (2016)	Technical indicators	RF	Gradient boosted trees (GBT), SVM, RF	Moroccan stock market
3. Rana et al. (2019)	Basic features	Decision tree classifier, Extra Tree classifier	LR, SVR, LSTM	Spanish stock market
4. Aloraini (2015)	Open prices	Pearson correlation coefficient (PCC), Spearman correlation, Euclidean distance, Manhattan distance, Search AIC score	Lasso estimate	11 equities in Saudi stock market
5. Kumar et al. (2016)	Basic features, Technical indicators	Pearson correlation, Spearman correlation, Relief algorithm, Random forest (RF)	PSVM	12 stock indices from different international markets
6. Alsubaie et al. (2019)	Technical indicators	Gain ratio, Relief algorithm, Correlation, Cost-based Naive Bayesian, Accuracy-based Naive Bayesian	9 different classifiers	99 stocks and TASI market in-dex
7. Li et al. (2022)	Technical indicators Fundamental indica- tors	PCC	Broad learning system	4 stocks from Shanghai Stock Exchange
8. Nabi et al. (2019)	Basic features	9 different methods	15 different classifiers	10 stocks from NASDAQ
9. Yuan et al. (2020)	Technical indicators, Fundamental indica-tors	RFE, RF	SVM RF ANN	Chinese A-share stocks
10. Botunac et al. (2020)	Basic features, Technical indicators	RFE, Linear regression, Decision Tree, RF	LSTM	Apple, Microsoft, Facebook
11. Shen et al. (2020)	Technical indicators	RFE PCA	LSTM	3558 Chinese stocks
12. Chen et al. (2017)	Technical indicators	Information gain	SVM	Chinese stock market indices
13. Sun et al. (2019)	Technical indicators	FSMRMR, CMIM	ARMA-GARCH-NN	US stock market
14. Singh et al. (2021)	Technical indicators, Fundamental indica-tors	PCA	6 different classifiers	505 stocks from S& P 500
15. Ampomah et al. (2020)	Basic features, Technical indicators	PCA	6 tree-based Classifiers	8 stocks from NYSE, NASDAQ, NSE
16. Siddique et al. (2019)	Basic features	PCA	SVR	TATA motors stock index
17. Iacomin (2015)	Technical indicators	PCA GA	SVM	16 Forex stocks from Bloomberg
18. Cai et al. (2012)	Basic features, Technical indicators	RBM	SVM	S& P 500 index
19. Das et al. (2019)	Technical indicators	PCA, Factor analysis (FA), Firefly optimization (FO), Genetic algorithm (GA), FO with GA	ELM, OSELM, RBPNN	4 different stock market indices
20. Qolipour et al. (2021)	Basic features, Technical indicators	PCA	Decision tree, RF, Gradient boosted tree (GBT)	2 stocks from Tehran stock exchange
21. Ampomah et al. (2021)	Technical indicators	PCA, LDA, FA	Gaussian Naïve Bayes (GNB)	7 stocks from NYSE, NASDAQ, NSE
22. Chen et al. (2020)	Basic features, Technical indicators	Information gain	FW-SVM	30 stocks
23. Gunduz etal. (2017)	Technical indicators	Gain ratio Relief algorithm	Gradient boosting ma-chine (GBM)	3 stocks in BIST market index
24. Kumar et al. (2021b)	Basic features, Technical indicators	PCA	ANN	3 stock indices
25. Tang et al. (2018)	historical relative re-turns	PCA	KNN	CSI 300 index
26. Barak et al. (2017)	Fundamental indica-tors	GA	Multiple classifiers	400 stocks
27. Farahani et al. (2021)	Technical indicators	GA	ANN	5 stock indices
28. Chong et al. (2017)	10 lagged returns	Autoencoder	DNN	38 stocks
29. Bhanja et al. (2022)	Technical indicators	Autoencoder	5 ML classifiers	2 market indices
30. Xie et al. (2021)	Fundamental indica-tors	Autoencoder	SVM	5 market indices
31. Dami et al. (2021)	Basic features	Autoencoder	LSTM	10 stocks
32. Gunduz (2021)	Technical indicators	Autoencoder	SVM LSTM	8 stocks

**Table 2:** Performance comparison based on target predictions, performance, and evaluation metrics

Study	Prediction target	Performance	Evaluation metrics
1. Haq et al. (2021)	Direction of daily stock prices	59.44 0.1030	Accuracy Matthews correlation coefficient (MCC)
2. Labiad et al. (2016)	Direction of 10 min ahead	90%	Accuracy
3. Rana et al. (2019)	Daily stock price	0.0151	RMSE
4. Aloraini (2015)	Daily open prices	0.15–0.63	predictive accuracy
5. Kumar et al. (2016)	Direction of one-day ahead	44.22–62.72	Accuracy
		44.64–100.00	Precision
		1.74–96.32	Recall
6. Alsubaie et al. (2019)	Direction of the stock returns	0.05–80.79	Accuracy,
		1.22–7.34	Cost of misclassification,
		0.00–0.16	Investment return percentage,
		0.49–1	Hit rate,
		1.04–1.93	Sharpe ratio,
		0.03–37.63	Number of bets
7. Li et al. (2022)	One-day ahead close price value	0.006	MSE,
		0.054	MAE,
		1.092	MAPE
		0.982	R2
		0.981	Adjusted R2
8. Nabi et al. (2019)	Direction of monthly price	100%	Accuracy
9. Yuan et al. (2020)	Direction of excess returns	52%	Accuracy, AUC
		53%	
10. Botunac et al. (2020)	Direction of close price	0.01606	MAE, MSE
		0.00046	
11. Shen et al. (2020)	Direction of stock price	0.93	Accuracy, Precision, Recall
		0.96	
		0.96	
12. Chen et al. (2017)	Direction of stock indices price	0.646–1.06	MAPE, RMSE
		0.0143–0.0239	
13. Sun et al. (2019)	Direction of Intraday market	1.404–1.443	Random cross-validation, Nearest-k cross-validation
		1.385–1.419	
14. Singh et al. (2021)	Direction of 10 days ahead	83.62%	Accuracy,
		85%	Precision,
		100%	Recall
15. Ampomah et al. (2020)	Direction of stock price	82%	Mean accuracy,
		85%	Precision,
		79%	Recall,
		82%	F1 score,
		84%	Specificity,
		90%	ROC curve
16. Siddique et al. (2019)	Next day close price	2.76	MAE,
		4.3	RMSE,
		0.63	MAPE
17. Iacomin (2015)	Direction of stock price	0.72	Accuracy
18. Cai et al. (2012)	Direction of one-day ahead close price	0.002 90.31	NMSE, Direction accuracy
19. Das et al. (2019)	Stock prices of 1,3,5,7,15,30 days in ad- vance	143.1104 0.308 121.8011 0.0002543 0.0080	RMSE, MAPE, MAE, Theil’s U, ARV
20. Qolipour et al. (2021)	Direction of stock return	0.947–1.0 0.993–1.0 0.880–1.0 0.993–1.0	Accuracy, Recall, Precision, AUC-ROC
21. Ampomah et al. (2021)	Direction of stock price	0.7188–0.8815 0.7014–0.8843 0.7763–0.8921 0.7916–0.9563	Accuracy, F1 score, Specificity, AUC
22. Chen et al. (2020)	Buy and sell signals	38.78 73.80%–98.84%	Accuracy, profit
23. Gunduz et al. (2017)	Daily returns	0.59 0.6	Accuracy F1 score
24. Kumar et al. (2021b)	1-day ahead close price	1.40E-03–9.34E-04 4.41E-05–3.07E-04 1.00E-04–7.44E-04 1.00E-04–7.44E-04	RMSE, MAPE, Theil’s inequality coefficient, ARV
25. Tang et al. (2018)	Next day return	79.60%	Hit rate
26. Barak et al. (2017)	Return	83.6%	Accuracy
27. Farahani et al. (2021)	Close price	13.499	MAE
28. Chong et al. (2017)	Direction of return	0.8224 0.9650 0.5931 0.0182	RMSE, NMSE, MAE, MI
29. Bhanja et al. (2022)	Direction of return	over 86%	Accuracy
30. Xie et al. (2021)	Direction prediction	53.3%–57.4%	Accuracy
31. Dami et al. (2021)	Returns prediction	0.022–0.039	MAE
32. Gunduz (2021)	Hourly direction	0.649 0.562	Accuracy F1 score

From Table 3, we can conclude that the correlation criteria, RF, PCA, and AE approaches are the most widely applied feature analysis techniques for various stock market predictions. For the datasets in Botunac et al. (2020); Kumar et al. 2016; Yuan et al. 2020; Labiad et al. 2016; Haq et al. 2021), RF provides good performance in terms of high accuracy and low error values. Meanwhile, PCA provides satisfactory results in Nabi et al. (2019); Shen and Shafiq 2020; Siddique and Panda 2019; Singh and Khushi 2021; Ampomah et al. 2020; Qolipour et al. 2021; Iacomin 2015; Ampomah et al. 2021; Das et al. 2019; Kumar et al. 2021b; Tang et al. 2018). Neural network-based models, and AEs have also been successfully applied for feature extraction (Chong et al. 2017; Bhanja and Das 2022; Xie and Yu 2021; Dami and Esterabi 2021; Gunduz 2021). Table 4 presents the most commonly applied ML predictive models in stock market analysis. RF and SVM are the most popular learning meth- ods because of their flexibility in classification and regression problems; they were respectively applied in 6 and 11 studies reviewed herein. Table 5 presents the cita- tion counts and journal indices of the reviewed studies.

**Table 3:** Feature selection/extraction techniques applied in the reviewed articles

Technique	Number of articles	Research articles
1. Correlation criteria	5	Alsubaie et al. (2019); Aloraini (2015); Li et al. (2022); Kumar et al. (2016); Nabi et al. (2019)
2. Distance criteria	1	Aloraini (2015)
3. Relief algorithm	3	Alsubaie et al. (2019); Kumar et al. (2016); Gunduz et al. (2017)
4. RFE	3	Botunac et al. (2020); Yuan et al. (2020); Shen and Shafiq (2020)
5. RF	5	Botunac et al. (2020); Kumar et al. (2016); Yuan et al. 2020; Labiad et al. (2016); Haq et al. (2021)
6. SVM	1	Haq et al. (2021)
7. Logistic regression	1	Haq et al. (2021)
8. Lasso estimate	1	Aloraini (2015)
9. RBM	1	Cai et al. (2012)
10. FSMRMR	1	Sun et al. (2019)
11. CMIM	1	Sun et al. (2019)
12. Information gain	2	Chen and Hao (2017); Chen and Hao (2020)
13. Gain ratio	2	Alsubaie et al. (2019); Gunduz et al. (2017)
14. PCA	11	Nabi et al. (2019), Shen and Shafiq (2020), Siddique and Panda (2019), Singh and Khushi (2021)
		Ampomah et al. (2020), Qolipour et al. (2021), Iacomin (2015), Ampomah et al. (2021)
		Das et al. (2019); Kumar et al. (2021b); Tang et al. (2018)
15. Autoencoder	5	Chong et al. (2017); Bhanja and Das (2022); Xie and Yu (2021); Dami and Esterabi (2021); Gunduz (2021)
16. LDA	1	Ampomah et al. (2021)
17. Factor analysis	2	Ampomah et al. (2021); Das et al. (2019)
18. Firefly optimization	1	Das et al. (2019)
19. Genetic algorithm	3	Das et al. (2019; Barak et al. (2017); Farahani and Hajiagha (2021)
20. FO with GA	1	Das et al. (2019)

**Table 4:** ML methods applied in the reviewed papers

ML methods	Number of articles	Research articles
1. Linear regression	1	Rana et al. (2019)
2. Naive bayes	3	Alsubaie et al. (2019); Nabi et al. (2019; Singh and Khushi (2021)
3. Gaussian Naive bayes	1	Ampomah et al. (2021)
(GNB)		
4. K-nearest neighbors	2	Chen and Hao (2017); Singh and Khushi (2021)
5. Lasso estimate	1	Aloraini (2015)
6. Broad learning system	1	Li et al. (2022)
(BLS)		
7. SVM	11	Cai et al. (2012; Alsubaie et al. (2019); Kumar et al. (2016); Nabi et al. (2019); Yuan et al. (2020); Labiad et al. (2016),
		Rana et al. (2019); Chen and Hao (2017); Siddique and Panda (2019); Singh and Khushi (2021); Iacomin (2015)
Tree-based ML methods	-	-
8. Decision tree	3	Nabi et al. (2019); Singh and Khushi (2021); Qolipour et al. (2021)
9. RF	6	Nabi et al. (2019); Yuan et al. (2020); Labiad et al. (2016); Singh and Khushi (2021); Ampomah et al. (2020); Qolipour et al. (2021)
10. Gradient boosted tree	2	Labiad et al. (2016); Qolipour et al. (2021)
Neural network methods	–	–
11. ELM	1	Das et al. (2019)
12. OSELM	1	Das et al. (2019)
13. RBPNN	1	Das et al. (2019)
14. Deep generative model	1	Haq et al. (2021)
15. ANN	2	Alsubaie et al. (2019); Yuan et al. (2020)
16. LSTM	3	Botunac et al. (2020); Shen and Shafiq (2020); Rana et al. (2019)

**Table 5:** Analysis of reviewed studies based on the number of citations and index of the journal

Study	Journal/Conference name	Number of citations	Index of the journal
1. Haq et al. (2021)	Expert Systems with Applications	11	Web of science
2. Labiad et al. (2016)	SITA	7	–
3. Rana et al., (2019)	CSAI	1	–
4. Aloraini (2015)	Evolving Systems	1	Web of science
		2	Scopus
5. Kumar et al. (2016)	Journal of Computational Science	27	Web of Science
		34	Scopus
6. Alsubaie et al. (2019)	IEEE Access	5	Web of science
		10	Scopus
7. Li et al. (2022)	IEEE Transactions on Circuits and Sys-tems	0	Web of science
		0	Scopus
8. Nabi et al. (2019)	Journal of Computer Science	0	Scopus
9. Yuan et al. (2020)	IEEE Access	15	Web of science
		27	Scopus
10. Botunac et al. (2020)	DAAAM proceedings	0	Scopus
11. Shen et al. (2020)	Journal of Big Data	15	Web of science
		36	Scopus
12. Chen et al. (2017)	Expert Systems with Applications	110	Web of science
		142	Scopus
13. Sun et al. (2019)	Expert Systems with Applications	12	Web of science
		16	Scopus
14. Singh et al. (2021)	Applied System Innovation	4	Web of science
		8	Scopus
15. Ampomah et al. (2020)	Information	29	Web of science
		28	Scopus
16. Siddique et al. (2019)	International Journal of Engineering and Advanced Technology	0	Scopus
17. Iacomin (2015)	ICSTCC	51	-
18. Cai et al. (2012)	CSAE	34	Scopus
19. Das et al. (2019)	Expert Systems with Applications	24	Scopus
20. Qolipour et al. (2021)	International Journal of Engineering	1	Web of science
		1	Scopus
21. Ampomah et al. (2021)	International Journal of Computing and Informatics	4	Web of science
		3	Scopus
22. Gunduz et al. (2020)	Turkish Journal of Electrical Engineering and Computer Sciences	8	Web of science
		13	Scopus
23. Chen et al. (2017)	Soft Computing	3	Web of science
		4	Scopus
24. Kumar et al. (2021b)	International Journal of Intelligent Sys-tems	2	Web of science
		4	Scopus
25. Tang et al. (2018)	International Journal of Computers Com-munications and Control	2	Web of science
		2	Scopus
26. Barak et al. (2017)	Information Fusion	30	Web of science
		38	Scopus
27. Farahani et al. (2021)	Soft Computing	6	Web of science
		7	Scopus
28. Chong et al. (2017)	Expert Systems with Applications	271	Web of science
		372	Scopus
29. Bhanja et al. (2022)	Innovations in Systems and Software En-gineering	1	Web of science
		1	Scopus
30. Xie et al. (2021)	Concurrency and Computation Practice and Experience	3	Web of science
		2	Scopus
31. Dami et al. (2021)	Multimedia Tools and Applications	5	Web of science
		4	Scopus
32. Gunduz (2021)	Financial Innovation	10	Web of science
		8	Scopus

**Fig. 3:**
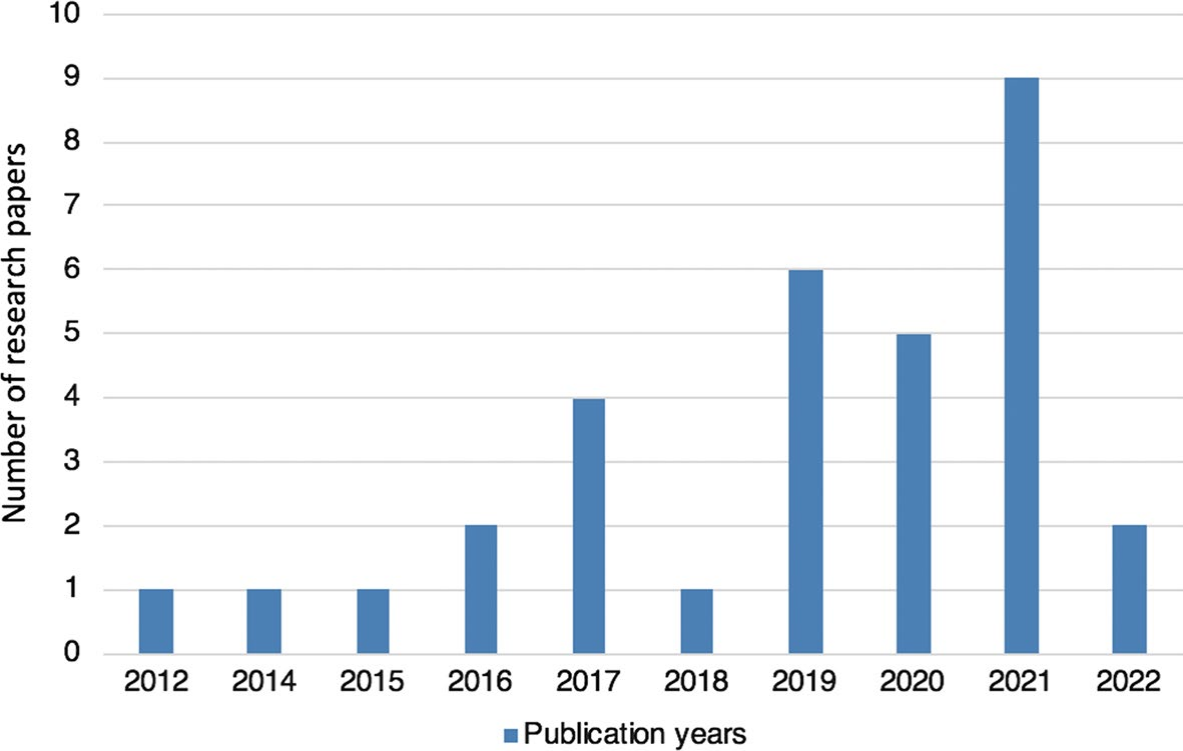
Analysis based on publication years

The analysis based on publication years is depicted in Fig. 3, which shows that the number of articles using feature selection/extraction methods became more popular in later years. In 2019 and 2021, six and nine articles on feature analysis for stock market prediction were published, and they covered all types of feature selection techniques: filter and wrapper methods (Alsubaie et al. 2019; Nabi et al. 2019), embedded methods (Haq et al. 2021; Rana et al. 2019), information theory-based methods (Sun et al. 2019), and feature extraction methods (Siddique and Panda 2019; Singh and Khushi 2021; Qolipour et al. 2021; Ampomah et al. 2021; Das et al. 2019; Kumar et al. 2021b; Xie and Yu 2021; Dami and Esterabi 2021; Gunduz 2021; Farahani and Hajiagha 2021).

## Limitations and future directions

In this survey, we covered research on feature analysis techniques applied to stock market analysis over the last 12 years. A significant number of studies have been conducted to prove the importance of feature reduction for stock datasets; however, we observed certain limitations. We noticed that only two papers (Aloraini 2015; Haq et al. 2021) studied an ensemble feature selection approach, which is a combination of three feature selection methods, whereas most existing studies employ a single approach for selecting critical features. Therefore, more research is needed to focus on the ensemble feature selection approach to obtain all features that affect predictions.

Regarding the types of features, most studies considered either basic features or technical or fundamental indicators. The number of studies that applied both basic features and technical indicators was lower than the number of studies that applied one type of feature. Therefore, further research is required to employ multiple fea- ture types from different categories. In Rana et al. (2019), closing price was found to be the most significant feature among the basic features; therefore, future work should consider applying closing price and technical indicators as input features to the model. In addition, three studies (Li et al. 2022; Yuan et al. 2020; Singh and Khushi 2021) that combined technical and fundamental in- dicators obtained accurate predictions. An interesting undertaking is to explore a combination of technical and fundamental features in the feature fusion process.

Another observation was that no study compared RF (feature selection) and PCA (feature extraction) methods that obtained the highest accuracy in the reviewed articles. Therefore, investigations into their performance differentiation on the same dataset need to be conducted.

We also noticed that most studies divided the experimental datasets into 70% training and 30% testing datasets to evaluate the performance of the predictive models. To consider a more practical problem of stock market forecasting, future research should us the sliding window method in splitting the sample into different groups of training and testing periods. The primary reason for using this method is that investors are always interested in the most recent stock trends but not in long-term historical data. Therefore, the predictive models should be updated periodically throughout the process. Future studies should examine the performance of the results based on different widths of the sliding window (one month, three months, six months, and one year) for the training and testing data because the movement of stock prices displays periodic behavior over various time scales.

## Conclusion

On the basis of our findings, we arrive to the following conclusions:

- The most frequently used feature selection and extraction approaches for vari- ous stock market applications were identified as correlation criteria, RF, PCA, and AE methods. In the last decade, the most popular ML methods have been RF and SVM.
- Most studies used individual types of features as inputs (basic features, technical indicators, or fundamental indicators) among structured-type inputs.
- Several of the reviewed studies demonstrated that feature selection and ex- traction improved the performance of the applied prediction methods.

We reviewed research papers that used a combination of feature analysis and ML models. Feature selection is an important aspect of the stock market forecasting, and accurate stock market predictions strongly depend on the selection of appropriate features. Therefore, researchers should focus on the use of various inputs and the application of feature reduction techniques to provide better feature sets for learning models.

## Data Availability

Not applicable.

## Abbreviations

ML: Machine learning
DL: Deep learning
EMH: Efficient market hypothesis
ARIMA: Autoregressive integrated moving average
LSTM: Long short term memory
SVM: Support vector machine
ANN: Artificial neural network
NSE: National stock exchange
RNN: Recurrent neural network
RFE: Recursive feature elimination
SVR: Support vector regression
OHLCV: Open, high, low, close, volume
CNN: Convolutional neural network
DBN: Deep belief network
DRL: Deep reinforcement learning
DNN: Deep neural network
DQN: Deep Q-network
RSI: Relative strength index
MACD: Moving average convergence divergence
PCC: Pearson correlation coefficient
PSVM: Proximal support vector machine
RR: Regression relief
RFE: Recursive feature elimination
RF: Random forest
MDA: Mean decrease accuracy
MDI: Mean decrease impurity
BLS: Broad learning system
RBM: Restricted Boltzmann machine
FSMRMR: Forward selection minimal redundancy maximal relevance
CMIM: Conditional mutual information maximization
PCA: Principal component analysis
MAE: Mean absolute error
RMSE: Root mean square error
MAPE: Mean absolute percentage error
LDA: Linear discriminant analysis
GNB: Gaussian Naive Bayes
